# Supplementary material for: Realizing symmetry-guaranteed pairs of bound states in the continuum in metasurfaces
Source: Nat Commun. 2022 Dec 7;13:7534. doi: 10.1038/s41467-022-35246-w (PMC9729201; doi:10.1038/s41467-022-35246-w)
Supplement: Supplementary file 1 — Supplementary Information [file 41467_2022_35246_MOESM1_ESM.pdf]

Supplementary material for realizing symmetry-guaranteed pairs of bound states in the continuum in metasurfaces

Chloe F. Doiron, Igal Brener, and Alexander Cerjan

1 Field Plots for two-dimensional band structure

Field plots for all modes at Γ for Figure 1b.

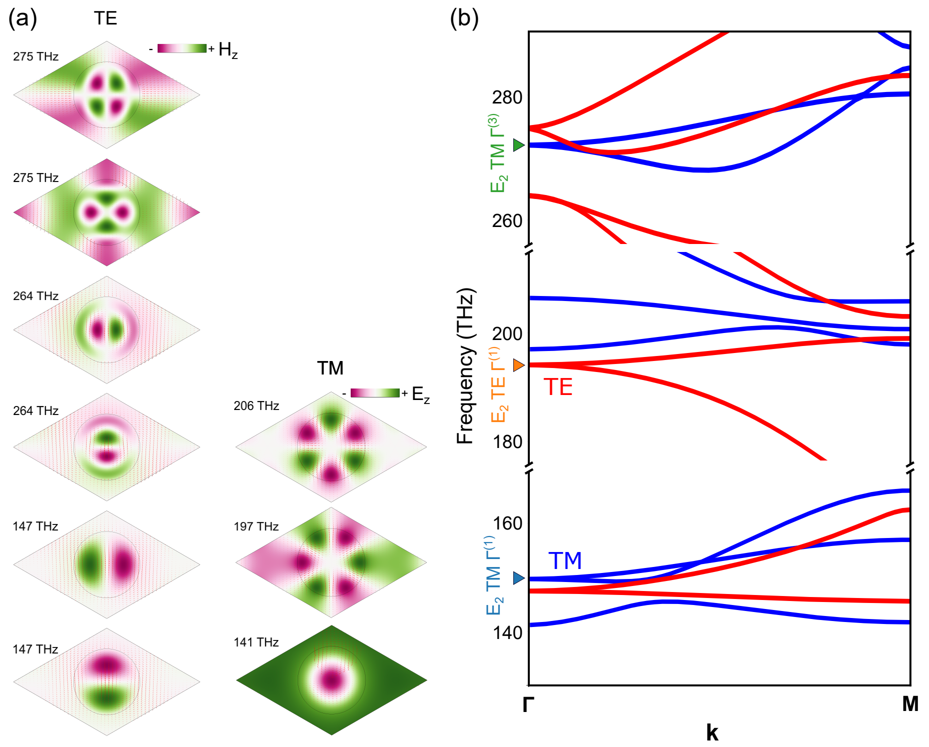

Figure S1: **Additional Field Profiles.** (**a**) Field profiles for all TE and TM modes at Γ-point. (**b**) Band structure reproduced from Figure 1b for the reader's convenience.

2 Expanded group theory discussion and graphical method

Several potential methods for breaking symmetry are proposed in Figure S2. These examples are representative of three practical aspects that should be considered when using group theory for designing symmetry-protected degenerate BICs. The three aspects are: (1) final symmetry of the perturbed field, (2) mode overlap with plane waves, and (3) path of symmetry degeneration.

(1) The symmetry of the perturbed field (Γ_{ψ_1}) is the direct product of the symmetry of the perturbation (Γ_v) and the reduced mode symmetry (Γ_{ψ_0}) leading to $\Gamma_{\psi_1} = \Gamma_v \times \Gamma_{\psi_0}$[1]. As a result of this direct product, the perturbed field and reduced mode symmetry are not required to belong to the same irreducible representation. This difference in irreducible representation makes it necessary to think of symmetry breaking in terms of space group symmetry of the reduced lattice instead of point group symmetry. In the example of deforming the resonators to squares (Figure S2b), the reduced lattice belongs to the cmm space group. These modes remain BICs since the perturbed fields (A_1 and A_2 irreducible representations) are even under C_2. Instead, if the resonators are deformed into half-square, half-circle resonators (Figure S2c) the lattice is reduced into the cm space group where the perturbed fields are no longer even under C_2 (A and B). This means that for the case of half-square, half-circle deformation the degenerate BICs are converted to two non-degenerate quasi-BICs capable of radiating to free space.

(2) When alternating rows of resonators are displaced (Figure S2d,e), the symmetry selection rules allow coupling to free space. Despite this allowance, the non-degenerate quasi-BICs are difficult to observe from free space. While the selection rules provide information on whether a mode can couple to free space, they contain no information on the coupling efficiency and *Q*-factor. As a result, the perturbed mode overlap with the far-field needs to be considered when choosing methods to break symmetry.

(3) Instead of performing a single symmetry breaking operation, symmetry breaking deformations can be viewed as a series of deformations that are separated by space group symmetry.

For example, by deforming the circular resonators to triangles (Figure S2f) the symmetry is reduced to $p31m$ lattice where the symmetry-protected degenerate BICs are reduced into symmetry-protected quasi-BICs with finite Q-factor. If this lattice is contracted along the vertical axis (Figure S2g) the symmetry is reduced even further to cm which lifts the degeneracy. By thinking of these symmetry breaking techniques as two distinct of symmetry breaking operations it is possible to tune Q-factor and frequency splitting independently.

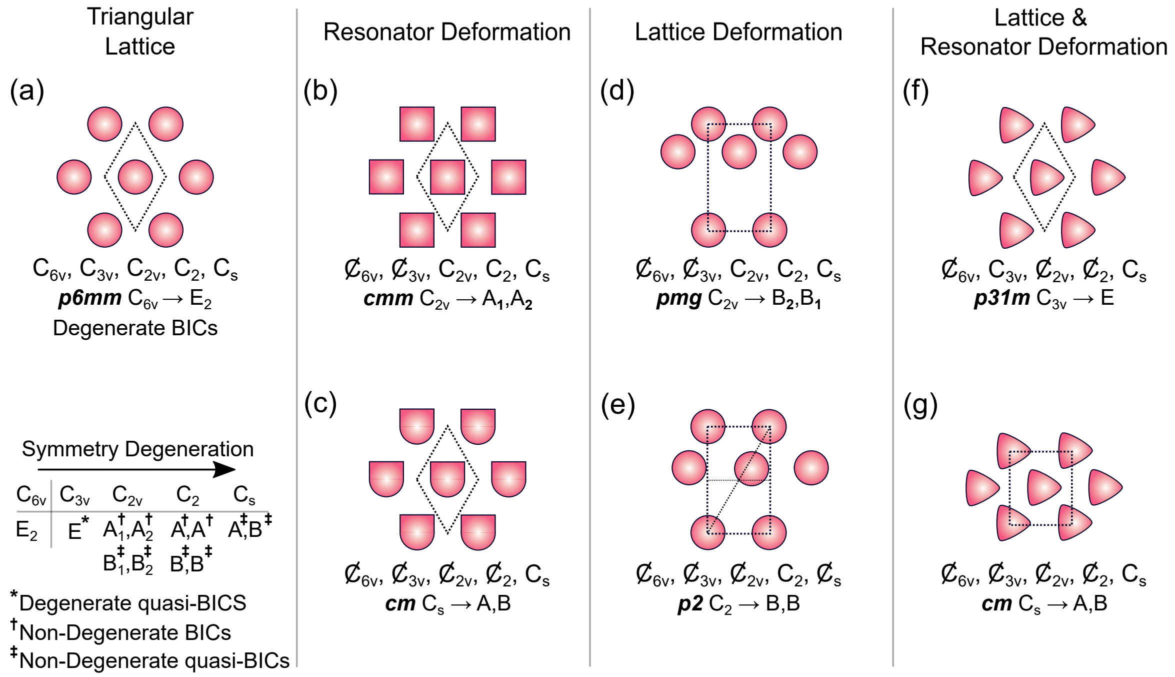

Figure S2: **Methods for symmetry breaking.** (**a**) Unperturbed triangular lattice of dielectric cylinders with rhombus primitive cell. The unperturbed lattice has C_{6v}, C_{3v}, C_{2v}, and C_{sv} symmetry. (**b**) By deforming the cylindrical resonators to squares C_{6v} and C_{3v} symmetries can be broken lifting the degeneracy, but C_{2v} symmetry is preserved reducing the lattice to the cmm space group with non-degenerate symmetry-protected BICs. (**c**) Deforming the resonators into half-square, half-circle breaks C_{2v} symmetry, reducing the space group to cm and converting the symmetry-protected degenerate BICs to non-degenerate quasi-BICs. (**d**) Translating alternating rows of resonators vertically can break C_{6v} and C_{3v} symmetries. The perturbed field symmetry is reduced to pmg, which converts the symmetry-protected BICs to non-degenerate quasi-BICs. Even if the translation is performed away from any symmetry lines (**e**) C_2 symmetry remains. This reduces the perturbed field symmetry to $p2$ which still results in two non-degenerate quasi-BICs. (**f**) A combination of resonator and lattice deformations can be used for symmetry breaking. By deforming the cylindrical resonators into triangles, C_{6v} and C_{2v} symmetries are broken degenerating the lattice to $p31m$ space group. For this space group, the symmetry-protected degenerate BICs are converted to degenerate quasi-BICs. (**g**) By contracting or dilating the lattice in one direction the triangular lattice is transformed into a cm lattice lifting the degeneracy. A combination of these symmetry deformations allows control of Q-factor and mode splitting.

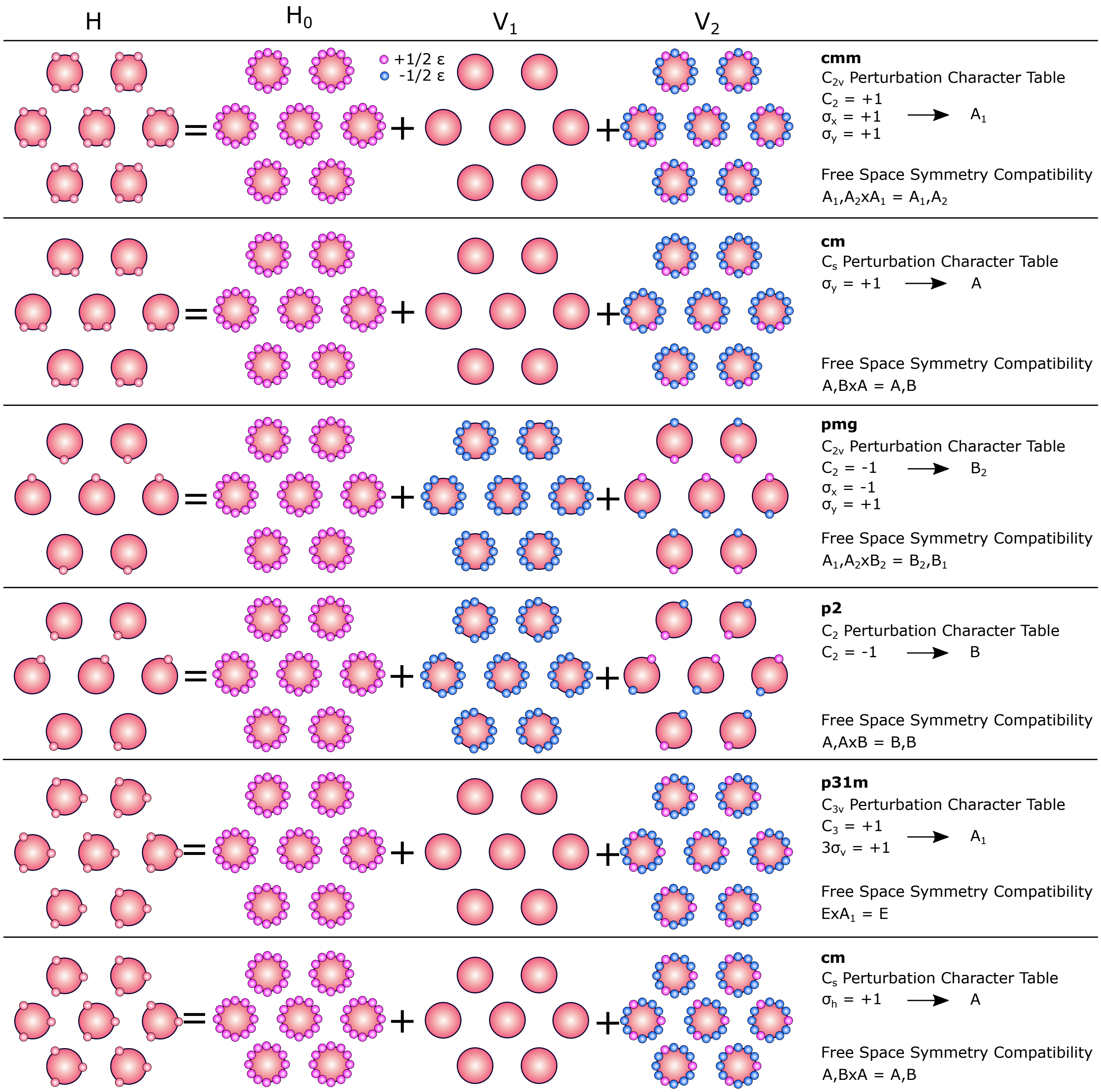

Figure S3: **Graphical derivation method.** Graphical derivation of selection rules for all lattice and resonator deformations shown in Figure S2

3 Additional reflectance spectra and SEM micrographs for engineering *Q*-factor and splitting

Additional spectra for S = 0.9 are shown in Figure S4. SEM micrographs for all geometries are presented in Figure S5.

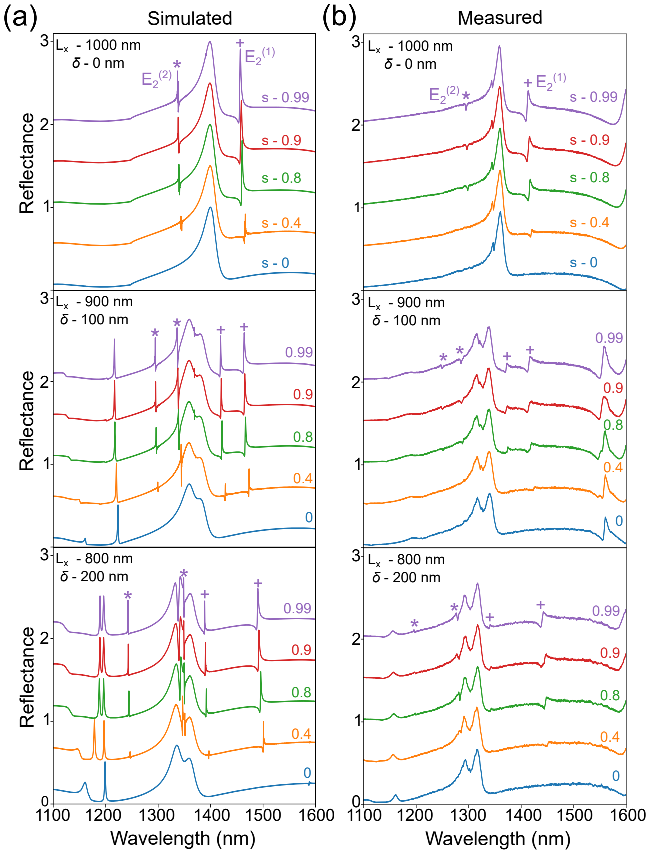

Figure S4: **Additional reflectance measurements.** Additional reflectance measurements for S = 0.9 presented with spectra shown in Figure 4

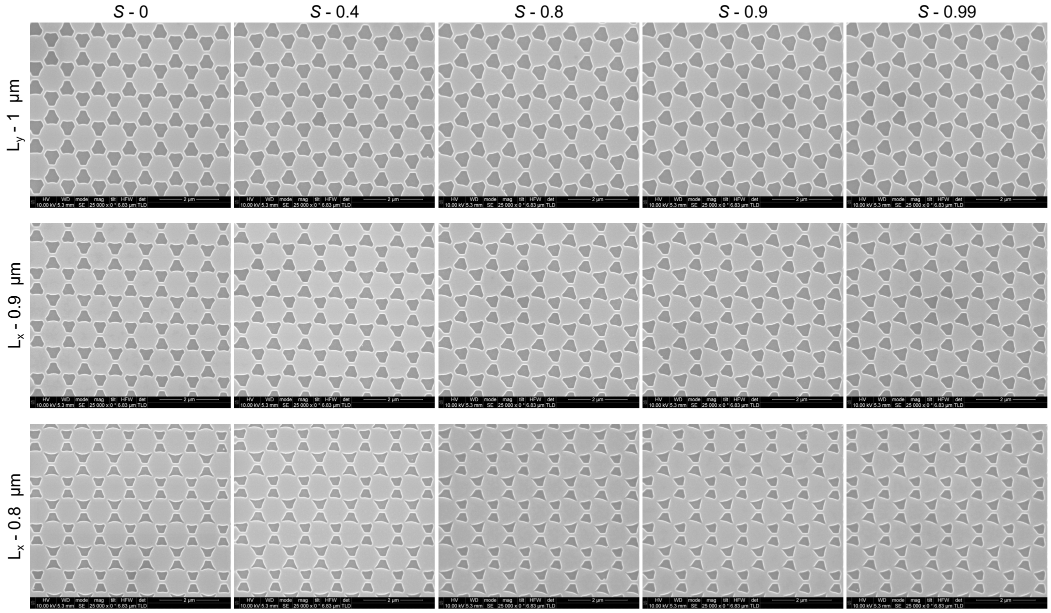

Figure S5: **SEM micrographs for tuning *Q*-factor and mode splitting.** SEM micrographs of all experimental samples from Figure 4 and Figure S4.

Experimental observation of symmetry-protected degenerate BICs using single resonator symmetry breaking parameter to control splitting and Q-factor

To illustrate a single symmetry breaking parameter converting the symmetry-protected degenerate BICs to non-degenerate quasi-BICs, we used half-circle, half-square resonator deformations. For this experimental demonstration, we fabricated silicon metasurfaces on fused silica substrates using standard nanofabrication techniques (see Methods). The fabricated metasurfaces had the following dimensions: period - $1000\,\mathrm{nm}$, thickness - $200\,\mathrm{nm}$, and bar width - $100\,\mathrm{nm}$. The diameter of the resonators was $790\,\mathrm{nm}$ when no symmetry breaking ($S = 0$) was present.

We performed full-wave electromagnetic eigenvalue simulations to understand the behavior of the symmetry-protected degenerate BICs as symmetry is smoothly broken. The wavelength and *Q*-factors for the $\mathrm{E}_2^{(1)}$ degeneracy are shown in Fig. S6a. As expected, when resonators are circles ($S = 0$) the modes are degenerate with resonance wavelengths $1459\,\mathrm{nm}$. The *Q*-factors

for both of these modes are $10^9 \approx \infty$ (limited by numerical precision) since the modes are BICs that are fully-decoupled from free space. The mode profiles for the $E_2^{(1)}$ BICs are presented in Fig. S6b. The symmetry of the field profiles show that both modes are even with respect to C_2 and are therefore symmetry-protected degenerate BICs. When the symmetry breaking parameter is non-zero the $p6mm$ (C_{6v}) lattice is deformed into a cm lattice which causes the symmetry-protected degenerate BICs to be converted to non-degenerate quasi-BICs since both C_2 and C_3 symmetries are broken. As the symmetry breaking parameter is increased the *Q*-factors decrease continuously and the mode spacing increases.

The fabricated metasurfaces had symmetry breaking parameters ranging from $S = 0$ to $S = 0.99$. Scanning electron micrographs of the metasurfaces are displayed in Fig. S6c. To characterize the ability to access the symmetry-protected degenerate BICs from free space, we performed normal incidence reflectance measurements using unpolarized light. Simulated and experimentally measured reflectance spectra are presented in Fig. S6a and S6b respectively. As predicted, when no symmetry breaking is present ($S = 0$) the $E_2^{(1)}$ and $E_2^{(2)}$ symmetry-protected degenerate BICs are not observable from free space. When the symmetry breaking parameter is increased to $S = 0.8$, the non-degenerate quasi-BICs can be seen in both simulated and measured spectra. Increasing the symmetry breaking even further to $S = 0.9$ leads to a mode splitting of only $2\,\mathrm{nm}$ in simulations with *Q*-factors 3440 and 5750. In comparison, in the measured spectrum we observed a $4\,\mathrm{nm}$ splitting and the observed *Q*-factors were 290 and 450. The increased splitting can be attributed to symmetry breaking due to fabrication imperfections, since the total symmetry breaking is the combination of symmetry breaking from design and imperfections. Increasing the symmetry breaking parameter even further to $S = 0.99$, increased the mode splitting to $7\,\mathrm{nm}$ and $10\,\mathrm{nm}$ for both simulated and measured spectra respectively. In comparison, the experimentally measured *Q*-factors only slightly decrease to 278 and 430. The experimentally measured decrease is significantly smaller than the two-fold reduction observed in simulations because the radiative component from imperfection induced symmetry breaking and non-radiative components limit the maximum attainable *Q*-factor. This experimental demonstration exemplifies the advantage of engineering symmetry-protected degenerate BICs. While the mode locations between simulations and

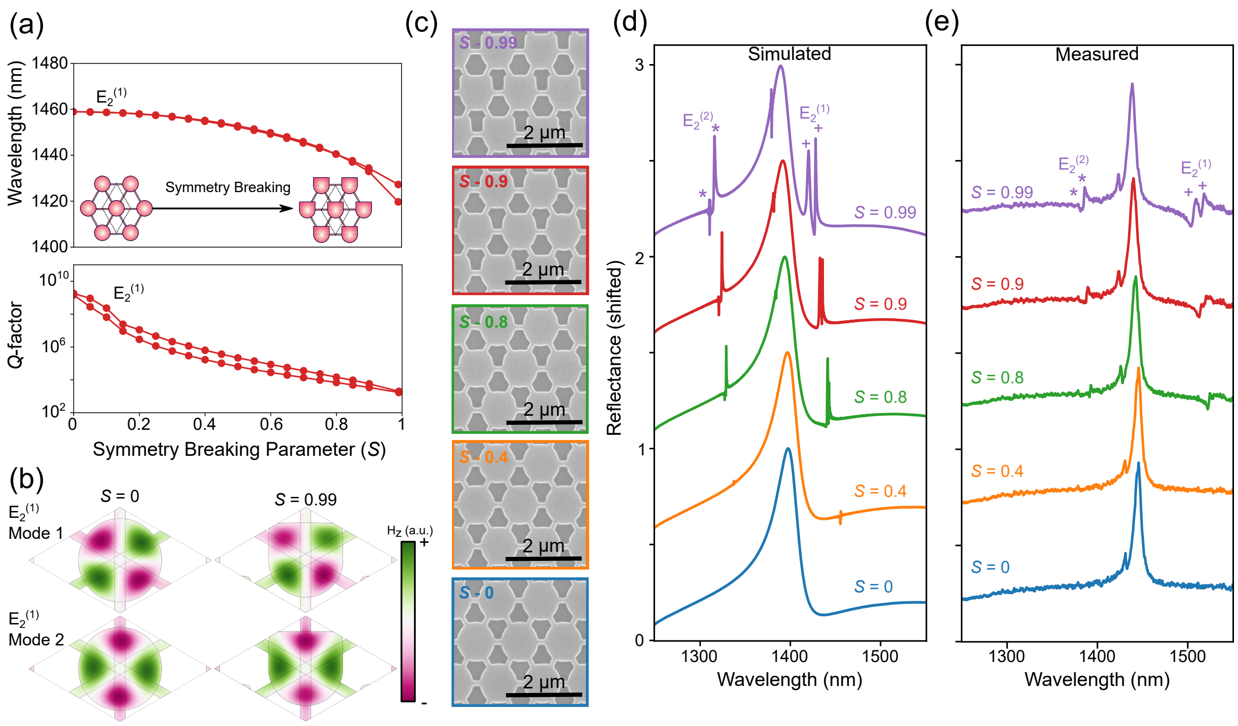

Figure S6: **Experimental demonstration of symmetry-protected degenerate BICs.** (**a**) Wavelength and Q-factor for the $E_2^{(1)}$ modes from full-wave simulations for half-square, half-circle silicon resonators in a triangular lattice an fused silica substrate. When the symmetry breaking parameter is zero (cylindrical resonators) the $E_2^{(1)}$ modes are degenerate BICs. As the symmetry breaking is increased the modes split and the Q-factor decreases as the modes become non-degenerate quasi-BICs. (**b**) Field profiles of H_z for $E_2^{(1)}$ modes with cylindrical resonators (S=0) and half-square, half-circle resonators (S=0.99). (**c**) Scanning electron micrographs of fabricated metasurfaces with symmetry breaking parameters from S=0 to S=0.99. Full-wave simulations (**d**) and experimental measurements (**e**) of reflectance of a silicon metasurface. As the symmetry breaking parameter is increased from zero, quasi-BICs appear from the $E_2^{(1)}$ and $E_2^{(2)}$ modes.

experiments differ by $90\,\mathrm{nm}$, the experimental mode splitting deviates from simulated results by only $3\,\mathrm{nm}$. Furthermore, the observed normalized splitting ($\frac{\Delta\omega}{\bar{\omega}}$) was only 5×10^{-3} corresponding to $0.9\,\mathrm{THz}$.

Resonator deformations to control mode spacing

Instead of using lattice deformations to break C_3 and control the mode spacing, it is possible to use resonator deformations. To illustrate this we performed full-wave eigenvalue simulations when deforming cylindrical resonators to square prisms as depicted in Fig. S7a. Through this deformation, C_6 and C_3 symmetries are broken resulting in a lattice that belongs to the cmm space group resulting in the symmetry-protected degenerate BICs becoming non-degenerate BICs. Correspondingly, as the symmetry breaking parameter is increased the mode splitting increases as shown in Fig. S7b, but since both modes are BICs the Q-factors remain $\approx \infty$ for all symmetry breaking parameters as confirmed in Fig. S7c.

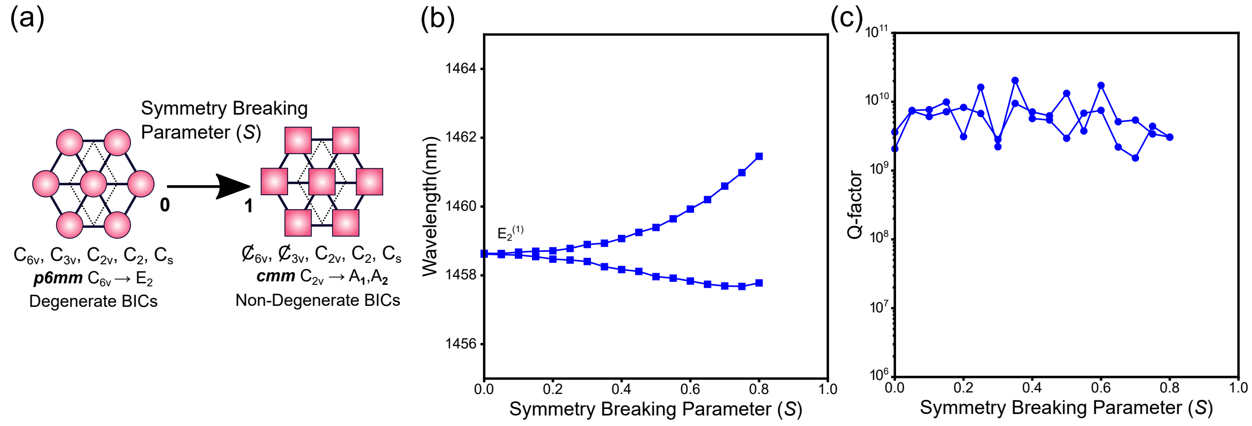

Figure S7: **Circle to square deformation to control splitting** (**a**) Schematic illustrating deforming the cylindrical resonators in a triangular lattice to square prism resonators. This breaks C_6 and C_3 symmetries reducing the the symmetry from a lattice belonging to the $p6mm$ space group to one belonging to the cmm space group causing the symmetry-protected degenerate BICs to become non-degenerate BICs. This can be observed from the mode wavelengths (**b**) and Q-factors (**c**) as a function of symmetry breaking. As the the cylindrical resonators are deformed into square prisms the degeneracy is lifted. But since the modes are non-degenerate BICs, the modes remain BICs with Q-factors $\approx \infty$ for all symmetry breaking parameters.

Lattice deformations to control mode spacing and Q-factors

To show a single lattice deformation capable of controlling mode spacing and Q-factors we performed full-wave eigenvalue simulations when the symmetry breaking operation was shifting alternating rows of resonators. A schematic of this symmetry breaking operation is presented in Fig. S8a. Shifting alternating rows of resonators causes all symmetries to be broken resulting in a lattice belonging to the $p1$ space group. This symmetry reduction, results in the symmetry-protected degenerate BICs in becoming non-degenerate quasi-BICs. This behavior is confirmed by the full-wave eigenvalue simulations. As the resonator deformation is increased from $0\,\mathrm{nm}$ the degeneracy is lifted (Fig. S8b) and the Q-factors (Fig. S8c) drop from infinity.

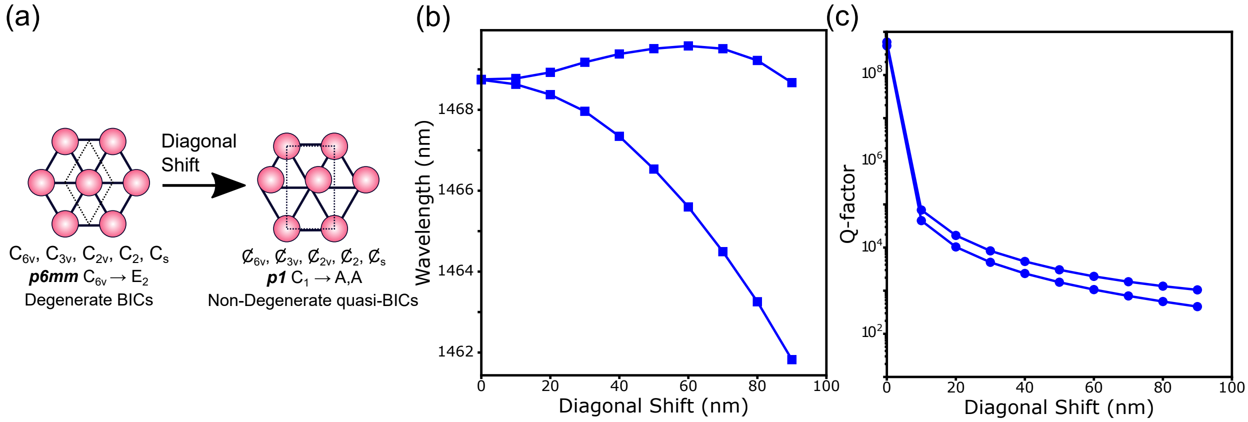

Figure S8: **Shifting alternating rows of resonators to control splitting and Q-factors** (**a**) Schematic illustrating shifting alternating rows of cylindrical resonators. This deformation breaks all symmetries reducing the the symmetry from a lattice belonging to the $p6mm$ space group to one belonging to the $p1$ space group causing the symmetry-protected degenerate BICs to become non-degenerate quasi-BICs. This effect can be observed in the wavelengths (**b**) and Q-factors (**c**). As expected, when the displacement is nonzero the degeneracy is lifted and the Q-factors become finite.

Effect of nonzero angle of incidence

In the experimental measurements, the angle of incidence was maintained less than $1°$. Nonzero angle of incidence results in symmetry breaking. To determine the effect of angle of incidence on symmetry reduction, we performed full-wave electromagnetic simulations of reflectance (Fig. S9a) when the resonator symmetry breaking (S) was 0.4 and lattice deformation (δ) of $0\,\mathrm{nm}$ for angles ranging from $0°$ to $3°$. From the calculated reflectance spectra, as the angle of incidence is increased the induced symmetry breaking results in the degeneracy being lifted and a reduction of the Q-factors. The calculated mode splitting (Fig. S9b) was recovered from the simulated reflectance curves by fitting the spectra with two Fano resonances. When the angle of incidence reaches $1°$ the mode splitting increases to $0.3\,\mathrm{nm}$. Increasing the angle of incidence further to $3°$ results in a splitting of $2.7\,\mathrm{nm}$. These calculated mode splittings are below the experimentally measured mode splittings for the half-circle, half-square resonator deformation case which observed splittings of $4\,\mathrm{nm}$ and $10\,\mathrm{nm}$ demonstrating that nonzero angle of incidence is not the largest contributor to symmetry breaking. For additional confirmation, we fit the experimentally measured reflectance measurements (Fig. S9c) of degenerate quasi-BICs when the resonator symmetry breaking (S) was 0.4 and lattice deformation (δ) of $0\,\mathrm{nm}$. Using a two Fano resonance fit resulted in a mode splitting of $1.5\,\mathrm{nm}$ representing an upper bound on the effect of angle of incidence since symmetry breaking can be caused by both nonzero angle of incidence and fabrication imperfections. This observed mode splitting would correspond to an angle of incidence of $2.2°$ for the case when nonzero angle of incidence was the only source of symmetry breaking.

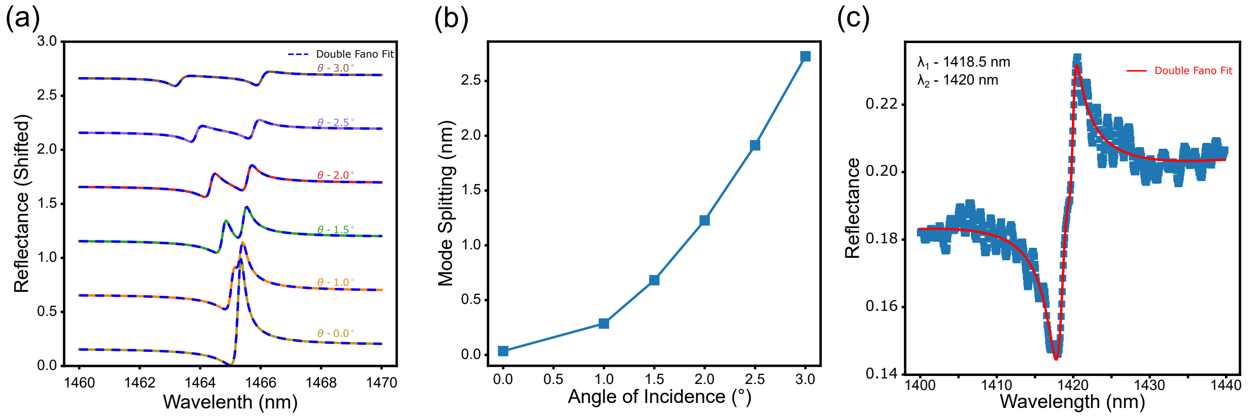

Figure S9: **Effect of nonzero angle of incidence** (**a**) Calculated reflectance spectra when the angle of incidence is nonzero. (**b**) To calculate the effect of angle of incidence on mode splitting, the calculated reflectance spectra were fit with two Fano resonances. As the angle of incidence was increased from $0°$ the degeneracy was lifted. (**c**) Representative experimentally measured reflectance spectra for the case when the modes are degenerate quasi-BICs (S$=0.4$, $\delta\ =0\,\mathrm{nm}$). Fitting the reflectance spectra with two Fano resonances results in a mode splitting of $1.5\,\mathrm{nm}$ providing a metric for the amount of symmetry breaking from nonzero angle of incidence and fabrication imperfections.

Effect of lattice contraction direction

To understand the role of lattice contraction direction on mode splitting and Q-factors we performed full-wave eigenvalue simulations for three lattice contraction directions: vertical, $63.4°$ diagonal, and horizontal. A schematic of the three contraction directions with respect to the triangular lattice is presented in Fig. S10a. For all cases, the C_2 symmetry breaking parameter (S) was 0.6. The calculated Q-factors and mode splittings (Fig. S10b) show that the choice of lattice contraction direction can have three quantitative effects. First, for a given mode splitting the mean Q-factors can depend strongly on contraction direction. For example, when the splitting was near $25\,\mathrm{nm}$, the mean Q-factors ($\overline{Q}$) for the vertical and horizontal cases are 2700 and 2730 respectively. In comparison, for the diagonal contraction case the calculated Q-factors were significantly higher with a $\overline{Q}$ of 3380. The second effect is the difference between the maximum (Q_{max}) and minimum (Q_{min}) Q-factors ($\Delta Q = |Q_{max} - Q_{min}|$). For the diagonal contraction case, the calculated ΔQ when the mode splitting is close to $25\,\mathrm{nm}$ is 1110. This difference reduces to 980 for the horizontal contraction case. The smallest ΔQ is obtained for the vertical contraction with a difference of 920. The final quantitative effect of lattice contraction direction is the degree of symmetry breaking for a given amount of lattice contraction. For the diagonal contraction case, the unit cell area has been reduced the most, but only achieves a mode splitting of around $25\,\mathrm{nm}$. While the unit cells are larger for the vertical and horizontal cases, they achieve mode splittings of $45\,\mathrm{nm}$ and $41\,\mathrm{nm}$ respectively. The reduced splitting in the diagonal case can be attributed to the fact that the diagonal lattice contraction is closer to preserving the triangular lattice (albeit with a scaled periodicity). If the lattice contraction direction only scaled the periodicity of the triangular lattice, C_3 symmetry would be preserved and the modes would remain degenerate. Combined, these three quantitative effects from lattice contraction direction demonstrate how the choice of symmetry breaking method can significantly affect device performance and provide insight into potential routes to optimize symmetry breaking operations.

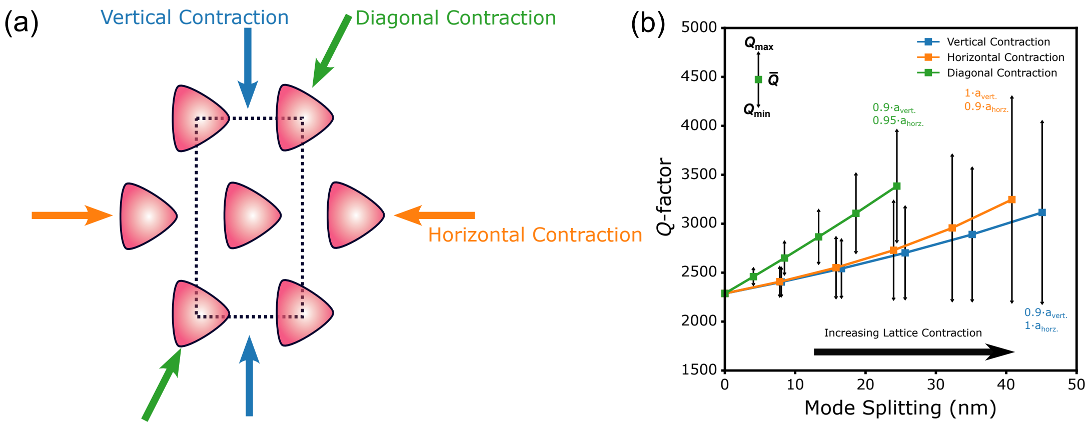

Figure S10: **Effect of lattice contraction direction on Q-factor** (**a**) Schematic denoting three different directions for contracting the triangular lattice. (**b**) Calculated Q-factors from full-wave eigenvalue simulations for vertical, horizontal, and diagonal lattice contractions. For equivalent mode splitting values the mean Q-factor ($\overline{Q}$) and difference between Q_{max} and Q_{min} can differ significantly.

1. Overvig, A. C., Malek, S. C., Carter, M. J., Shrestha, S. & Yu, N. Selection rules for quasibound states in the continuum. *Physical Review B* **102**, 035434 (2020).
